# Bleeding Risk in Nonvalvular Atrial Fibrillation Patients Receiving Direct Oral Anticoagulants and Warfarin: A Systematic Review and Meta-Analysis of Observational Studies

**DOI:** 10.1055/s-0040-1714918

**Published:** 2020-07-13

**Authors:** Yimin Pearl Wang, Rohan Kehar, Alla Iansavitchene, Alejandro Lazo-Langner

**Affiliations:** 1 Division of Hematology, Department of Medicine, Western University, London, Ontario, Canada; 2 Library Services, London Health Sciences Centre, London, Ontario, Canada; 3 Department of Epidemiology and Biostatistics, Western University, London, Ontario, Canada

**Keywords:** atrial fibrillation, direct oral anticoagulants, bleeding, observational studies, meta-analysis

## Abstract

**Introduction** In randomized trials in atrial fibrillation (AF) patients on direct oral anticoagulants (DOACs) have a lower risk of bleeding compared with warfarin. However, data from randomized trials may not extrapolate to general population. We aimed to determine the risk of bleeding in patients on DOACs in observational studies.

**Materials and Methods** Observational studies from 1990 to January 2019 were included. A pooled effect hazard ratio (HR) was calculated with a random effects model using the generic inverse variance method. Subgroup analyses according to previous anticoagulants exposure, study type, funding source, and DOAC type (direct thrombin inhibitors vs. factor Xa inhibitors) were conducted.

**Results** A total of 35 studies comprising 2,356,201 patients were included. The average pooled HR for observational data was 0.78 (95% confidence interval [CI] 0.71, 0.85). There were no statistically significant differences in pooled HR by previous exposure to anticoagulants, DOAC type (direct thrombin vs. factor Xa inhibitors), study type, and funding source. Among patients receiving factor Xa inhibitors, patients on apixaban had a lower risk of bleeding compared with warfarin (HR 0.60, 95% CI 0.50, 0.71, *p* < 0.001) in contrast to those on rivaroxaban (HR 0.98, 95% CI 0.91, 1.06, *p* = 0.60).

**Conclusion** In observational studies, AF patients on DOACs experience less bleeding events compared with warfarin; however, apixaban and dabigatran, but not rivaroxaban, have a lower risk of bleeding than warfarin.

## Introduction

Atrial fibrillation (AF) is one of the most common sustained cardiac arrhythmias in the general population with a prevalence of 1 to 2% 1 and is often associated with an increased risk of death, stroke, bleeding, and other thromboembolic events. 2 AF has a large influence on health care as a result of hospitalization, stroke, and other related costs. Due to the rising prevalence of AF, proper care is needed to reduce burden and health care cost. 3

Based on the North American guidelines, there are two types of AF, valvular and nonvalvular depending on whether there are valvular abnormalities such as the presence of mitral stenosis or artificial valves. 4 The standard oral anticoagulant therapy administered to nonvalvular AF patient has typically been vitamin K antagonists, particularly, warfarin. 1 In recent years, direct oral anticoagulants (DOACs)—including direct thrombin inhibitors (DTI) and direct factor Xa (FXa) inhibitors—have become an alternative to warfarin due to its relatively stable pharmacokinetics 5 resulting in no need for laboratory monitoring and less pharmacological interactions compared with warfarin. 6

Randomized trials comparing warfarin and DOACs showed comparable effectiveness without significant additional major bleeding risk. 5 In 2011, ROCKET-AF, a noninferiority randomized control trial (RCT) of 14,264 subjects showed no statistically significant difference in bleeding risk between rivaroxaban and warfarin (hazard ratio [HR] 1.04, 95% confidence interval [CI] 0.90, 1.20). 7 However, bleeding events in RCTs may differ from those in daily use due to the routine exclusion of patients with a higher risk of bleeding from many studies. 6 7

Therefore, we aimed to assess bleeding risk between DOACs and warfarin in AF patients in observational studies and we also sought to determine differences between patients that were or were not previously on anticoagulants and whether early bleeding risk (within 3 months) is different than late (after 3 months) bleeding risk.

## Materials and Methods

### Search Strategy

A systematic literature search was conducted in the OVID MEDLINE and Embase electronic databases. The search queries were developed with a combination of Medical Subject Headings and keywords including hemorrhage, atrial fibrillation, warfarin, and DOAC ( Supplementary Material, Appendix 1 ). The search strategy was adapted for each database. Additional relevant studies that met the inclusion criteria were identified through bibliographies of relevant retrieved articles.

**Fig. 1:**
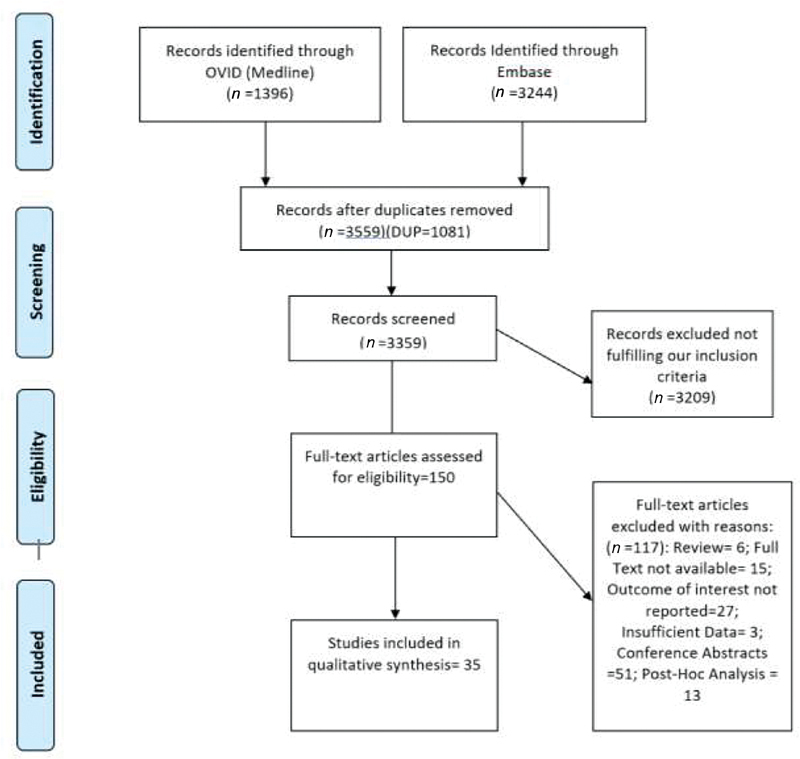
PRISMA (Preferred Reporting Items for Systematic Reviews and Meta-Analyses) flow diagram summarizing the identification process of relevant studies.

### Criteria for Including Studies

We primarily aimed to include all observational studies, including cohort studies and case–control studies evaluating a DOAC and warfarin. Review articles, editorials, commentaries, conference publication, and letters to the editor were excluded. Studies were eligible if they were published between January 1990 and June 2018 (subsequently extended to January 2019), included patients aged 18 or older, and were available in English. Additionally, RCTs comparing a DOAC with warfarin were as well retrieved and included in secondary analyses to evaluate their influence on the estimates of interest.

### Outcome Measures

The primary outcome was major bleeding risk. Secondary outcome was clinically relevant nonmajor bleeding (CRNMB). All studies must have used an established or validated definition of major bleeding, such as the one proposed by the International Society of Thrombosis and Haemostasis (ISTH) or similar 8 9 ( Supplementary Material, Appendix 2 ).

**Fig. 2:**
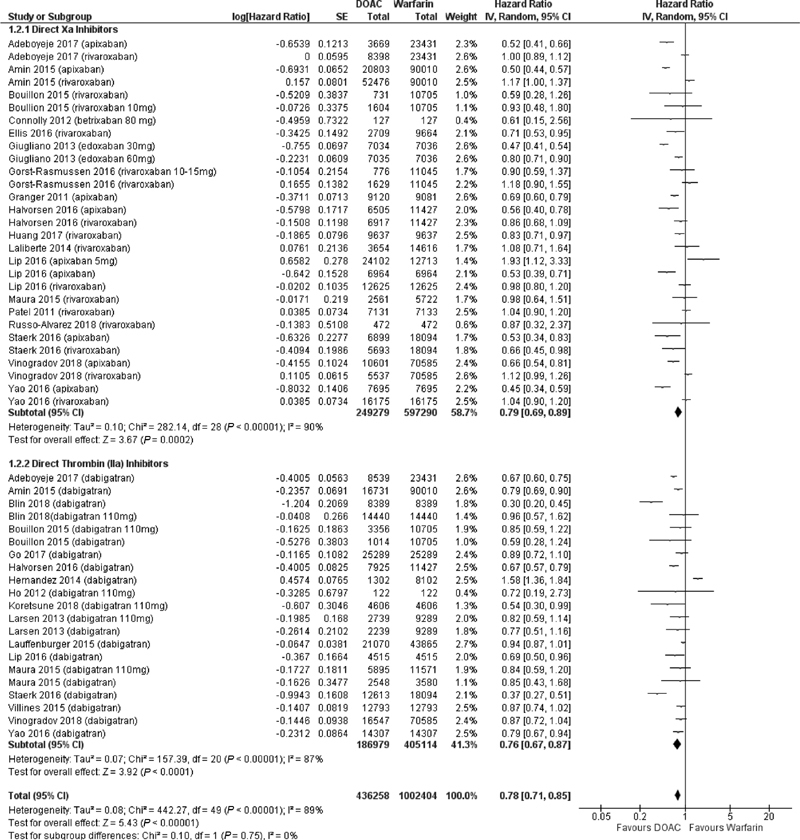
Comparison of bleeding risk between direct oral anticoagulants and warfarin stratified by drug class.

### Study Selection

Two reviewers independently examined and retrieved studies by assessing the study title and abstract. Cohen's kappa coefficient 10 was computed after each level of screening to assess the agreement level. Articles were included for full-text screening if the two reviewers agreed. Disagreements were resolved in conjunction with a third reviewer by discussion and consensus. Assessments of study outcomes and study population were made before studies were included in this review.

### Assessment of Quality and Data Extraction

Two reviewers independently extracted data from selected studies using a standardized data extraction form. Data regarding number of patients in each intervention, total bleeding events, and effect estimates of bleeding risk were collected. Additional data such as duration of follow-up, loss to follow-up, and patient enrollment were also recorded. Quality of the studies was evaluated using the Newcastle–Ottawa Scale 11 for observational studies and the Jadad scale for RCTs. 12

### Statistical Analysis

An overall pooled HR and its 95% CI for each outcome were calculated using a random effects model. 13 Sensitivity analyses were conducted by study type, previous anticoagulant exposure, individual agent, type of DOAC (DTI vs. FXa inhibitors), and source of funding. All analyses were performed using Review Manager Version 5.3 (The Nordic Cochrane Centre, The Cochrane Collaboration, Copenhagen, Denmark). The generic inverse variance method 14 was used to calculate an overall average effect estimate. The standard errors were obtained using the CI given by the studies and log HRs were calculated using the logarithms of the studies' HRs. Heterogeneity was assessed using the chi-squared test and the Higgins' *I* ^2^ test. 15

### Registration

The protocol for the systematic review was registered on PROSPERO International Prospective Register of Systematic Reviews with the number CRD42019120468.

## Results

### Study Selection and Characteristics

The initial literature search identified 3,359 potentially eligible citations. After primary screening, 150 articles were eligible for full-text review and there were 35 studies 7 16 17 18 19 20 21 22 23 24 25 26 27 28 29 30 31 32 33 34 35 36 37 38 39 40 41 42 43 44 45 46 47 48 49 50 including 2,356,201 patients that met the inclusion criteria and presented relevant data regarding major bleeding and clinically relevant bleeding ( Fig. 1 ). The studies were primarily observational including 25 retrospective cohorts, 6 prospective cohorts, and 4 RCTs ( Table 1 ). The focus of our review was primarily based on observational data. Results including RCTs are included in the Supplementary Material .

**Table 1:** Characteristics of included studies

First author (y)	Type of study	Total number of patients	Type of DOACs	Bleeding definition	Funding
Adeboyeje et al (2017)	Retrospective cohort	44,057	Dabigatran, apixaban, and rivaroxaban	ICD	Health insurance
Amin et al (2017)	Prospective cohort	180,020	Dabigatran, apixaban, and rivaroxaban	ISTH	Pharmaceutical
Bengtson et al (2017)	Retrospective cohort	145,666	Dabigatran	ICD	Research grant
Blin et al (2019)	Prospective cohort	103,101	Dabigatran	ISTH	Pharmaceutical
Bouillon et al (2015)	Retrospective cohort	17,410	Dabigatran and rivaroxaban	ICD	No funding
Connolly et al (2013)	Randomized control trial	508	Betrixaban	ISTH	Pharmaceutical
Denas et al (2017)	Retrospective cohort	13,480	Dabigatran, apixaban, and rivaroxaban	ICD	Research grant
Ellis et al (2016)	Retrospective cohort	18,429	Dabigatran and rivaroxaban	ICD	Pharmaceutical
Giugliano et al (2013)	Randomized control trial	21,105	Edoxaban	ISTH	Pharmaceutical
Go et al (2017)	Retrospective cohort	50,578	Dabigatran	ICD	Government
Gorst-Rasmussen et al (2016)	Retrospective cohort	13,450	Rivaroxaban	ICD	Nonprofit
Graham et al (2019)	Retrospective cohort	897,888	Dabigatran, apixaban, and rivaroxaban	ICD	Government
Granger et al (2011)	Randomized control trial	18,201	Apixaban	ISTH	Pharmaceutical
Halvorsen et al (2017)	Retrospective cohort	32,675	Dabigatran, apixaban, and rivaroxaban	ICD	Pharmaceutical
Hernandez et al (2015)	Retrospective cohort	9,404	Dabigatran	ICD	Nonprofit
Ho et al (2012)	Prospective cohort	244	Dabigatran	ISTH	Academia
Huang et al (2018)	Retrospective cohort	19,274	Rivaroxaban	ICD	Government
Jacobs et al (2016)	Prospective cohort	5,254	Dabigatran, apixaban, and rivaroxaban	ICD	No funding
Koretsune et al (2019)	Retrospective cohort	9,212	Dabigatran	ISTH	Pharmaceutical
Laliberté et al (2014)	Retrospective cohort	18,270	Rivaroxaban	ICD	Pharmaceutical
Larsen et al (2013)	Prospective cohort	13,914	Dabigatran	ICD	No funding
Larsen et al (2014)	Retrospective cohort	11,315	Dabigatran	ICD	Nonprofit
Lauffenburger et al (2015)	Retrospective cohort	64,935	Dabigatran	ICD	Academia
Li et al (2017)	Retrospective cohort	153,880	Apixaban	ICD	Pharmaceutical
Lip et al (2016)	Retrospective cohort	48,208	Dabigatran, apixaban, and rivaroxaban	ICD	No funding
Lip et al (2016)	Retrospective cohort	15,115	Dabigatran, apixaban, and rivaroxaban	ICD	Pharmaceutical
Maura et al (2015)	Prospective cohort	32,807	Dabigatran and rivaroxaban	ICD	No funding
Norby et al (2017)	Retrospective cohort	133,740	Rivaroxaban	ICD	Research Grant
Patel et al (2011)	Randomized control trial	14,264	Rivaroxaban	ISTH	Pharmaceutical
Russo-Alvarez et al (2018)	Retrospective cohort	944	Rivaroxaban	ICD	No funding
Staerk et al (2017)	Retrospective cohort	43,299	Dabigatran, apixaban, and rivaroxaban	ICD	Nonprofit
Villines et al (2015)	Retrospective cohort	25,586	Dabigatran	ICD	Pharmaceutical
Vinogradova et al (2018)	Prospective cohort	103,270	Dabigatran	ICD	Research grant
Wu et al (2019)	Prospective cohort	344	Dabigatran, rivaroxaban	Other	Not provided
Yao et al (2016)	Retrospective cohort	76,354	Dabigatran, apixaban, and rivaroxaban	ICD	Research grant

Abbreviations: DOAC, direct oral anticoagulant; ICD, International Classification of Disease; ISTH, International Society of Thrombosis and Haemostasis.

### Study Quality

Key quality features and quality assessment of each study are included in the Supplementary Material . All RCTs were deemed as good quality by the Jadad scale with a score of 4 or 5 ( Supplementary Material, Appendix 3 ). Using the Newcastle–Ottawa Scale, the prospective and retrospective cohorts varied as a result of differences in follow-up time and presences of outcome at the start of the study, but were in general adequate ( Supplementary Material, Appendix 4 ).

### Major Bleeding Events for DOACs Compared with Warfarin

**Fig. 3:**
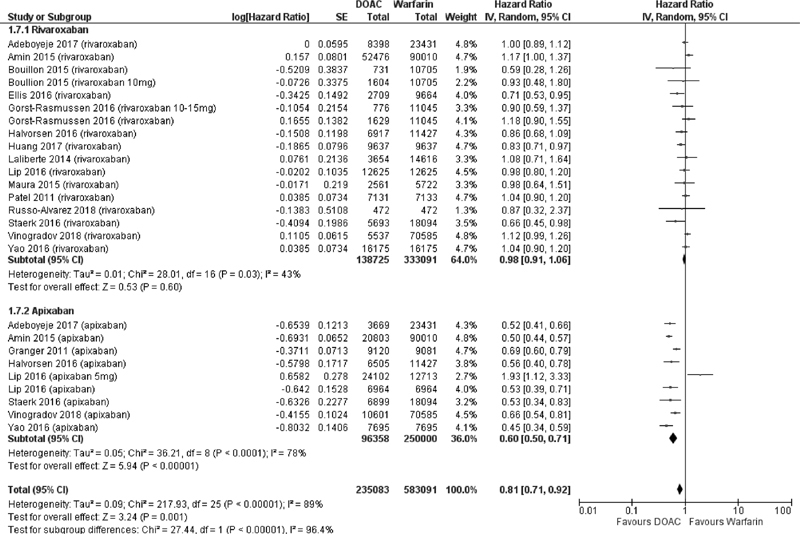
Comparison of bleeding risk between direct factor Xa inhibitors and warfarin stratified by individual agent.

Overall, patients on DOACs were less likely to experience a bleeding event compared with warfarin (HR 0.78, 95% CI 0.71, 0.85, *p* < 0.001). The results were consistent when analyzing patients receiving DTIs or FXa inhibitors (DTI: HR 0.76, 95% CI 0.67, 0.87; FXa inhibitors: HR 0.79, 95% CI 0.69, 0.89) ( Fig. 2 ). However, among patients receiving FXa inhibitors there was a significant difference in the risk of bleeding according to individual drug. Among patients receiving rivaroxaban the risk of bleeding was similar to warfarin (HR 0.98, 95% CI 0.91, 1.06, *p* = 0.60), whereas in those receiving apixaban there was a 40% reduction in the risk of bleeding compared with warfarin (HR 0.60, 95% CI 0.50, 0.71, *p* < 0.001) ( Fig. 3 ).

### Bleeding Risk according to Previous Anticoagulant Exposure

Three studies 18 36 43 reported information according to previous anticoagulant exposure. The overall pooled HR was 0.68 (95% CI 0.55, 0.82, *p* < 0.001) in favor of patients on DOACs ( Supplementary Material, Appendix 5 ). In the subgroup analysis according to previous anticoagulant use the risk of bleeding was lower for DOACs compared with warfarin in both experienced population (HR 0.70, 95% CI 0.51, 0.96) and naive patients (HR 0.64, 95% CI 0.47, 0.87). However, heterogeneity was moderate to high among both subgroups.

### Short-Term and Long-Term Major Bleeding Risk

Only one study by Norby et al 43 showed that patients switching from warfarin to rivaroxaban had a higher risk of gastrointestinal bleeding in the first 90 days after switching compared with the risk after 90 days.

### Clinically Relevant Nonmajor Bleeding Risk

Two studies reported data on CRNMB. The pooled average HR was 0.64 (95% CI 0.47, 0.87, *p* < 0.001) in favor of DOACs ( Supplementary Material, Appendix 6 ) with high statistical heterogeneity. Not enough studies were included in the meta-analysis for subgroup analysis for DTIs and FXa inhibitors using only data from observational studies. Using data from both RCT and observational studies, patients receiving DOACs had a lower risk of CRNMB (HR 0.73; 95% CI 0.62, 0.86, *p* < 0.001) in comparison to warfarin ( Supplementary Material, Appendix 7 ).

### Effect Estimate Stratified by Study and Funding Type

There were no significant differences between effect estimates stratified by study and funding type. The pooled HR of only observational data was 0.80 (95% CI 0.73, 0.88, *p* < 0.001) and for RCT data was 0.72 (95% CI 0.53, 0.97, *p* = 0.03) ( Supplementary Material, Appendix 8 ). There was no statistical difference in effect estimates by study source of funding. Studies that received pharmaceutical funding had an HR of 0.77 (95% CI 0.67, 0.88, *p* < 0.001), while studies that received government and research aid had an HR of 0.81 (95% CI 0.73, 0.91, *p* < 0.001) ( Supplementary Material, Appendix 9 ).

## Discussion

This review and meta-analysis of observational studies including over 2.3 million patients showed that overall, DOACs showed a lower risk of major bleeding and CRNMB compared with warfarin. Most importantly, although the pooled effect estimate did not differ between the two drug classes, namely DTIs and FXa inhibitors, among patients receiving the latter there was a significant difference between individual agents with patients on apixaban having a significantly lower risk of bleeding compared with warfarin in contrast to patients on rivaroxaban who had a similar risk. The average pooled HR did not differ by study and funding type.

These findings are consistent with data from RCTs. Previous systematic reviews and meta-analyses of only RCTs showed results similar to the present study; however, the results were not statistically significant due to the presence of statistical heterogeneity. 51 Ruff et al found that on average, AF patients in RCTs that were on DOACs are less likely to experience a major bleed in comparison to those who were on warfarin (relative risk 0.83, 95% CI 0.73, 1). 51 Concerns regarding power within RCTs of smaller sample size may arise because in all RCTs, major bleeding was considered as a secondary or safety outcome. Moreover, results in RCTs may not reflect the bleeding events ratios in clinical practice.

A recent systematic review of real-world bleeding risk in clinical settings had similar results and in contrast with that study our review does provide quantifiable effect estimates. 52 Both studies found no significant difference in bleeding between AF patients taking rivaroxaban and AF patients taking warfarin. Dabigatran and apixaban both were associated with a lower risk for bleeding. Previous independent studies have also come to similar conclusions, but in contrast, the present study provides the largest number of patients included to date and several subgroup analyses that have not been previously conducted, including the association of previous exposure to anticoagulants with the risk of bleeding. 53 54 Taken together, these results provide independent confirmation of the safety profile of DOACs in AF patients and raise new and important questions to define future projects.

Our study has some limitations. First, we were unable to ascertain short-term and long-term bleeding risks because included studies varied in their reporting and follow-up. Although we aimed to determine differences of early versus late bleeding events, all studies but only one reported one bleeding risk instead of stratifying major bleeding risks before and after the 3-month period. 43 Norby et al 43 stratified their bleeding risk before and after 90 days of taking anticoagulants. Their study showed that patients switching to rivaroxaban had a higher risk of gastrointestinal bleeding in the first 90 days after switching compared with the risk after 90 days. This is an important question that needs more studies as it may lead to potential clinical interventions addressing such risk. Second, several studies did not specify loss to follow-up or duration of follow-up which limits their quality and more importantly, validity. Third, similar to previous systematic reviews and meta-analyses of RCTs, 51 52 statistical heterogeneity was present in our study. Studies differed mostly in data collection, outcome definition, and location. Some studies used clinical data while others relied on insurance claim data. This would introduce inconsistency in reporting across studies as congruency between clinical and claims data ranges from 65 to over 90%. 55 Clinical data tends to have more details regarding patient characteristics such as laboratory results, medications, and comorbidities. There were differences in bleeding definitions used. Some studies used different revisions of the International Classification of Disease. All RCTs 7 21 24 28 and four observational studies 17 19 31 34 used the definition provided by ISTH. The differences in definition could result in the inclusion of a variation of bleeding types that were classified as major bleed. Study location ranged from North America to Europe to Asia where level of care, education, and the social economic status of the AF patients included may differ. Among the studies, different dosages were either pooled together or reported separately which would introduce more heterogeneity in interpretation. This is particularly the case for dabigatran. This variability could account for some of the insignificant differences between predetermined subgroup analyses. As a result of these inconsistencies across studies, the overall pooled estimate should be interpreted with caution. Fourth, of the included studies, there were only four that looked at differences in bleeding risk in naive and experienced AF patients. With DOACs being an alternative to warfarin, many AF patients are introduced to a DOAC for the first time or switched from warfarin to a DOAC. In those patients being switched from warfarin to a DOAC it is possible that preexisting conditions that increase the risk of bleeding might have been detected during the period in which patients were on warfarin, thus resulting in medical interventions to deal with such conditions and potentially resulting in a lower bleeding risk after the patients are switched. More attention should be focused on the bleeding risk between naive and experienced patients. Finally, studies that reported CRNMB were scarce as well. CRNMB still results in an increased level of care and medical interventions which is a matter of concern for patients, clinicians, and health systems.

In summary, this review showed that DOACs appeared to be associated with a lower bleeding risk compared with warfarin. Although there were no differences in bleeding outcomes between the DTIs and FXa inhibitors, among the latter, those patients on apixaban seem to have less bleeding events in comparison to warfarin while those on rivaroxaban have the same risk. Our study highlights the need for standardized definitions to better define outcomes, better data collection, and proper reporting for clinically relevant outcome assessments.
