# Supplementary material for: Bleeding Risk in Nonvalvular Atrial Fibrillation Patients Receiving Direct Oral Anticoagulants and Warfarin: A Systematic Review and Meta-Analysis of Observational Studies
Source: TH Open. 2020 Jul 13;4(3):e145–52. doi: 10.1055/s-0040-1714918 (PMC7358046; doi:10.1055/s-0040-1714918)
Supplement: Supplementary file 1 — Supplementary Material [file 10-1055-s-0040-1714918-s200010.pdf]

Supplementary Material

Appendix 1 Search Strategy

Database: Ovid MEDLINE(R) ALL <1946 to January 28, 2019>

1 exp HEMORRHAGE/ (312374)
2 (h?emorrhag$ or bleed$ or rebleed$ or re-bleed$).mp. (472129)
3 (clotting or (blood$ adj clot$)).tw,kw. (26736)
4 or/1-3 (574057)
5 atrial fibrillation/ or atrial flutter/ (51647)
6 ((AVAF or AF) and (atrial or flutter)).tw,kw. (22630)
7 ((atrial$ or auricular$) adj3 (fibrillation$ or flutter$)).tw,kw. (65846)
8 or/5-7 (76532)
9 4 and 8 (8889)
10 (vitamin$ K or warfarin$).ti.orexp *warfarin/ or exp *Vitamin K/ (22149)
11 exp *anticoagulants/ or (anticoagula$ or anti-coagulant$ or anti-coagulation$ or anti-thrombin$ or antithrombin$ or NOAC or DOAC or NOACs or DOACs or acenocoumarol or coumarin or phenprocoumon or sintrom or sinthrome or jantoven or nicoumalone or dicoumarol or dicumarol or phenindione or dabigatran or ximelagatran or apixaban or rivaroxaban or edoxaban or betrixaban or idraparinux).ti. (124203)
12 exp warfarin/ or exp Vitamin K/ or (VKA or VKAs or (vitamin$ adj2 K) or warfarin$ or Waran or Coumadin$ or Marevan or bristol-myerssquibb).tw,kw. (50332)
13 exp anticoagulants/ or (anticoagula$ or anti-coagulant$ or anti-coagulation$ or anti-thrombin$ or antithrombin$ or NOAC or DOAC or NOACs or DOACs).tw,kw. (255173)
14 (TSOAC or TOAC or TSOACs or TOACs or (thrombin$ adj2 inhibitor$) or (factor$ adj3 Xa adj3 inhibitor$) or (non-VKA or non-VKAs or ((non-vitamin$ or anti-vitamin$) adj2 K) or ((nonvitamin$ or antivitamin$) adj2 K))).tw,kw. (8967)
15 (acenocoumarol or coumarin or phenprocoumon or sintrom or sinthrome or jantoven or nicoumalone or dicoumarol or dicumarol or phenindione or dabigatran or ximelagatran or apixaban or rivaroxaban or edoxaban or betrixaban or idraparinux).tw,kw. (20691)
16 or/13-15 (266026)
17 9 and ((10 and 16) or (11 and 12)) (3755)
18 Comparative Study/ or (vs$1 or vs or versus or compar$).tw. (6314669)
19 ((multicenter$ or multi-center$).tw. and ((between or each or both or either or than or across) adj2 group$1).tw,kw.) or groups.tw. or group$1.ab. /freq = 2 (2419101)
20 or/18-19 (7381116)
21 17 and 20 (2300)
22 (non-valvular$ or nonvalvular$ or NVAF).tw,kw. (3850)
23 early diagnosis/ or ((recenc$ or recent$ or new$2) adj3 (detect$ or identif$ or diagnos$)).tw,kw. (225555)
24 (early or newly$ or initiat$ or untreated$ or un-treated$ or not-treat$ or non-treat$ or ((user$1 or onset or patient$) adj3 new$2)).tw,kw. (2282434)
25 na?ve$.tw,kw. (78386)
26 or/22-25 (2471394)
27 9 and (12 and 16) and 20 and 26 (1164)
28 21 or 27 (2405)
29 limit 28 to english language (2233)
30 limit 29 to yr = "1990 - 2019" (2227)
31 30 not (exp Animals/ not (Human/ and exp Animals/)) (2218)
32 limit 31 to "all adult (19 plus years)" (1171)
33 limit 31 to "all child (0 to 18 years)" (46)
34 31 not (33 not (32 and 33)) (2218)
35 (mice or rat or rats or cat$1 or cattle$1 or dog$1 or goat$1 or horse$1 or rabbit$1 or sheep$1 or swine$1 or pig$1 or canine$1 or feline$1 or porcine$ or calf).ti. (1702356)
36 (pediatr$ or paediatr$ or child$ or adolescent$ or infan$ or newborn$ or neonat$).ti. (1270075)
37 34 not (or/35-36) (2217)
38 prospective studies/ or exp cohort studies/ or longitudinal studies/ or cohort.tw. or longitudinal.tw. or (prospective$ and cohort).mp. or ((random$ or multicenter$ or multi-center$ or center$ or multicentr$ or multi-centr$ or centr$ or pilot$ or

population$ or observational or validation or phase$) adj3 (trial or study)).mp. or clinical trial.tw. orexp clinical trial/ or double-blind method/ or random allocation/ or prospectively.tw. or prospective.tw. or (case$ adj5 study).mp. or case-base.mp. or case-comparison.mp. or case-compeer.mp. or case-control.mp. or case-referent.mp. or matched case.mp. or nested case.mp. or (match$ adj3 control$).tw. or case-control studies/ or retrospective.tw. or retrospectively.tw. or retrospective studies/ or follow$ up.mp. or follow-up studies/ or cross sectional.mp. or epidemiologic studies/ or epidemiologic$.tw. or observational.tw. or controls.tw. or control group.tw. or case series.tw. or trial.tw. or comparative studies/ or time factors/ or (outcome$ or mortality or risk$ or survival or morbidity or study or matched or report or registry).ti. or patient satisfaction/ or survival analysis/ or socioeconomic factors/ or treatment outcome/ or "outcome assessment (health care)"/ or comorbidity/ or (study adj2 design).tw. or (case$ and control$).tw. or (case$ and series).tw. orexp Evaluation Studies as Topic/ (9391836)
39 37 and 38 (1887)
40 limit 37 to "reviews (best balance of sensitivity and specificity)" (706)
41 37 and (meta-analysis or systematic review).tw. (257)
42 or/40-41 (711)
43 39 not 42 (1396)

Database: Embase Classic + Embase <1947 to 2019 January 30>
Search Strategy:

1 exp bleeding/ (911123)
2 (h?emorrhag$ or bleed$ or rebleed$ or re-bleed$).tw,kw. (630958)
3 (clotting or (blood$ adj clot$)).tw,kw. (43679)
4 or/1-3 (1115630)
5 exp *atrial fibrillation/ or *heart atrium flutter/ or *heart atrium fibrillation/ (69259)
6 ((AVAF or AF) and (atrial or flutter)).tw,kw. (46707)
7 ((atrial$ or auricular$) adj3 (fibrillation$ or flutter$)).tw,kw. (119049)
8 or/5-7 (124739)
9 4 and 8 (20663)
10 (vitamin$ K or warfarin$).ti. (19127)
11 (anticoagula$ or anti-coagulant$ or anti-coagulation$ or anti-thrombin$ or antithrombin$ or NOAC or DOAC or NOACs or DOACs or acenocoumarol or coumarin or phenprocoumon or sintrom or sinthrome or jantoven or nicoumalone or dicoumarol or dicumarol or phenindione or dabigatran or ximelagatran or apixaban or rivaroxaban or edoxaban or betrixaban or idraparinux).ti. (60410)
12 *warfarin/ or (VKA or VKAs or (vitamin$ adj2 K) or warfarin$ or Waran or Coumadin$ or Marevan or bristol-myerssquibb).tw,kw. (76689)
13 exp *anticoagulant agent/ or (anticoagula$ or anti-coagulant$ or anti-coagulation$ or anti-thrombin$ or antithrombin$ or NOAC or DOAC or NOACs or DOACs).tw,kw. (351733)
14 (TSOAC or TOAC or TSOACs or TOACs or (thrombin$ adj2 inhibitor$) or (factor$ adj3 Xa adj3 inhibitor$) or (non-VKA or non-VKAs or ((non-vitamin$ or anti-vitamin$) adj2 K) or ((nonvitamin$ or antivitamin$) adj2 K))).tw,kw. (13675)
15 *dabigatran/ or *rivaroxaban/ or *apixaban/ or (acenocoumarol or coumarin or phenprocoumon or sintrom or sinthrome or jantoven or nicoumalone or dicoumarol or dicumarol or phenindione or dabigatran or ximelagatran or apixaban or rivaroxaban or edoxaban or betrixaban or idraparinux).tw,kw. (34411)
16 or/13-15 (366548)
17 9 and ((10 and 16) or (11 and 12)) (6496)
18 exp comparative study/ or (vs$1 or vs or versus or compar$).tw. orcm.fs. (8351449)
19 (multicenter$ or multi-center$).tw.and ((between or each or both or either or than or across) adj2 group$1).tw,kw. (21554)
20 or/18-19 (8354357)
21 17 and 20 (4459)
22 warfarin/cm or *antivitamin K/cm or *dabigatran/cm or *rivaroxaban/cm or *apixaban/cm (7056)
23 9 and 22 (2375)
24 (non-valvular$ or nonvalvular$ or NVAF).tw,kw. (7136)
25 early diagnosis/ or ((recenc$ or recent$ or new$2) adj3 (detect$ or identif$ or diagnos$)).tw,kw. (395324)
26 (early or newly$ or initiat$ or untreated$ or un-treated$ or not-treat$ or non-treat$ or ((user$1 or onset or patient$) adj3 new$2)).tw,kw. (3179024)
27 na?ve$.tw,kw. (126434)
28 or/24-27 (3459785)
29 9 and (12 and 16) and 20 and 28 (2495)

30 21 or 23 or 29 (5650)
31 limit 30 to english language (5401)
32 limit 31 to yr = "1990 - 2019" (5393)
33 32 not ((exp animal/ or nonhuman/) not exp human/) (5370)
34 limit 33 to (adult <18 to 64 years> or aged <65+ years >) (2148)
35 limit 33 to (embryo <first trimester> or infant <to one year> or child <unspecified age >) (19)
36 33 not (35 not (34 and 35)) (5365)
37 (mice or rat or rats or cat$1 or cattle$1 or dog$1 or goat$1 or horse$1 or rabbit$1 or sheep$1 or swine$1 or pig$1 or canine$1 or feline$1 or porcine$ or calf).ti. (2070397)
38 (pediatr$ or paediatr$ or child$ or adolescent$ or infan$ or newborn$ or neonat$).ti. (1612424)
39 36 not (or/37-38) (5356)
40 (cohort$1 or longitudinal or ((random$ or multicenter$ or multi-center$ or center$ or multicentr$ or multi-centr$ or centr$ or pilot$ or population$ or observational or validation or phase$) adj3 (trial or study)) or clinical trial or prospectively or prospective or (case$ adj5 study) or case-base or case-comparison or case-compeer or case-control or case-referent or matched case or nested case or (match$ adj3 control$) or retrospective or retrospectively).tw. or follow$ up.mp. or cross sectional.mp. or observational.tw. or controls.tw. or control group.tw. or case series.tw. or trial.tw. or epidemiologic$.tw. or (outcome$ or mortality or risk$ or survival or morbidity or study or matched or report or registry).ti. or (study adj2 design).tw. or case study/ or clinical study/ or cohort analysis/ or comparative study/ or cross-sectional study/ or double blind procedure/ or exp case control study/ or exp clinical trial/ or exp comparative study/ or expcontrolled study/ or follow up/ or intervention study/ or longitudinal study/ or major clinical study/ or observational study/ or observational study.mp. or open study/ or outcomes research/ or pilot study/ or prevention study/ or prospective study/ or randomization/ or retrospective study/ or validation study/ [Multipatient Studies Filter EMBASE] (14480248)
41 39 and 40 (4369)
42 limit 39 to "reviews (best balance of sensitivity and specificity)" (1575)
43 39 and (meta analysis/ or "systematic review"/ or systematic review.tw.) (606)
44 or/42-43 (1603)
45 41 not 44 (3244)

Appendix 2 Definitions for Bleeding Events

Definition of Clinically Relevant Nonmajor Bleeding in AF and nonsurgical VTE Studies according to the International Society on Thrombosis and Haemostasis

1. Any signs or symptom of hemorrhage (i.e., more bleeding than would be expected for a clinical circumstance, including bleeding found by imaging alone) that does not fit the criteria for the ISTH definition of major bleeding but does meet at least one of the following criteria:
   (1) Requiring medical intervention by a healthcare professional.
   (2) Leading to hospitalization or increased level of care.
   (3) Prompting a face to face evaluation.

Definition of Major Bleeding in Clinical Investigations of Antihemostatic Medicinal Products in Nonsurgical Patients according to the International Society on Thrombosis and Haemostasis

1. Fatal bleeding, and/or
2. Symptomatic bleeding in a critical area or organ, such as intracranial, intraspinal, intraocular, retroperitoneal, intra-articular, or pericardial, or intramuscular with compartment syndrome, and/or
3. Bleeding causing a fall in hemoglobin level of 20/gL (1.24 mmol/L) or more or leading to a transfusion of twoor more units of whole blood or red cells.

Appendix 3 Jadad Score for Randomized Control Trials

Study (Year)	Randomization (max 2)	Double blind (max 1)	Withdrawal/Dropout (max 2)	Jadad Score (max 5)
Connolly (2012)	2	0	2	4
Giugliano (2013)	2	1	1	4
Granger (2011)	2	1	2	5
Patel (2011)	2	1	1	4

Appendix 4 Newcastle–Ottawa Assessment for Observational Studies

First author (y)	Type of study	Selection (max 4)	Comparability (max 2)	Outcome (max 3)
Adeboyeje (2017)	Retrospective cohort	***	*	***
Amin (2017)	Prospective cohort	***	*	**
Bengtson (2016)	Retrospective cohort	****	**	***
Blin (2018)	Prospective cohort	***	**	***
Bouillon (2015)	Retrospective cohort	****	**	***
Denas (2017)	Retrospective cohort	***	*	**
Ellis (2016)	Retrospective cohort	***	*	***
Go (2017)	Retrospective cohort	***	*	**
Gorst-Rasmussen (2016)	Retrospective cohort	***	*	***
Graham (2019)	Retrospective cohort	***	*	**
Halvorsen (2016)	Retrospective cohort	***	*	**
Hernandez (2014)	Retrospective cohort	***	*	***
Ho (2012)	Prospective cohort	***	*	***
Huang (2017)	Retrospective cohort	***	*	***
Jacobs (2016)	Prospective cohort	***	*	***
Koretsune (2018)	Retrospective cohort	***	*	**
Laliberte (2014)	Retrospective cohort	***	*	**
Larsen (2013)	Prospective cohort	****	*	***
Larsen (2014)	Retrospective cohort	****	**	***
Lauffenburger (2015)	Retrospective cohort	****	*	***
Li (2016)	Retrospective cohort	****	*	**
Lip (2016)	Retrospective cohort	****	*	**
Lip (2016)	Retrospective cohort	***	*	**
Maura (2015)	Prospective cohort	****	*	***
Norby (2017)	Retrospective cohort	****	**	**
Russo-Alvarez (2018)	Retrospective cohort	***	*	***
Staerk (2016)	Retrospective cohort	****	**	***
Villines (2015)	Retrospective cohort	***	*	***
Vinogradova (2019)	Prospective cohort	****	*	**
Wu (2018)	Prospective cohort	***	*	**
Yao (2016)	Retrospective cohort	****	*	**

Study or Subgroup	log[Hazard Ratio]	SE	DOAC Total	Warfarin Total	Weight	Hazard Ratio IV, Random, 95% CI
**1.1.1 Experienced AF Population**						
Bengston 2016 (experienced)	-0.8675	0.3072	13937	63460	7.1%	0.42 [0.23, 0.77]
Larsen 2014 (experienced d110)	-0.1863	0.1326	2038	8504	15.4%	0.83 [0.64, 1.08]
Larsen 2014 (experienced d150)	-0.5276	0.1382	2214	8504	15.0%	0.59 [0.45, 0.77]
Norby 2017 (experienced)	0.0392	0.232	11845	43904	9.9%	1.04 [0.66, 1.64]
**Subtotal (95% CI)**			**30034**	**124372**	**47.5%**	**0.70 [0.51, 0.96]**
Heterogeneity: Tau² = 0.06; Chi² = 8.81, df = 3 (P = 0.03); I² = 66%						
Test for overall effect: Z = 2.21 (P = 0.03)						
**1.1.2 Naive AF Population**						
Bengston 2016 (naive)	-0.9943	0.3139	18981	37707	6.9%	0.37 [0.20, 0.68]
Larsen 2014 (naive d110)	-0.0943	0.1125	2035	14126	16.6%	0.91 [0.73, 1.13]
Larsen 2014 (naive d150)	-0.4005	0.1196	4018	14126	16.2%	0.67 [0.53, 0.85]
Norby 2017 (naive)	-0.5978	0.1754	32495	89400	12.8%	0.55 [0.39, 0.78]
**Subtotal (95% CI)**			**57529**	**155359**	**52.5%**	**0.64 [0.47, 0.87]**
Heterogeneity: Tau² = 0.07; Chi² = 11.53, df = 3 (P = 0.009); I² = 74%						
Test for overall effect: Z = 2.84 (P = 0.004)						
**Total (95% CI)**			**87563**	**279731**	**100.0%**	**0.68 [0.55, 0.82]**
Heterogeneity: Tau² = 0.05; Chi² = 20.34, df = 7 (P = 0.005); I² = 66%						
Test for overall effect: Z = 3.88 (P = 0.0001)						
Test for subgroup differences: Chi² = 0.17, df = 1 (P = 0.68), I² = 0%						

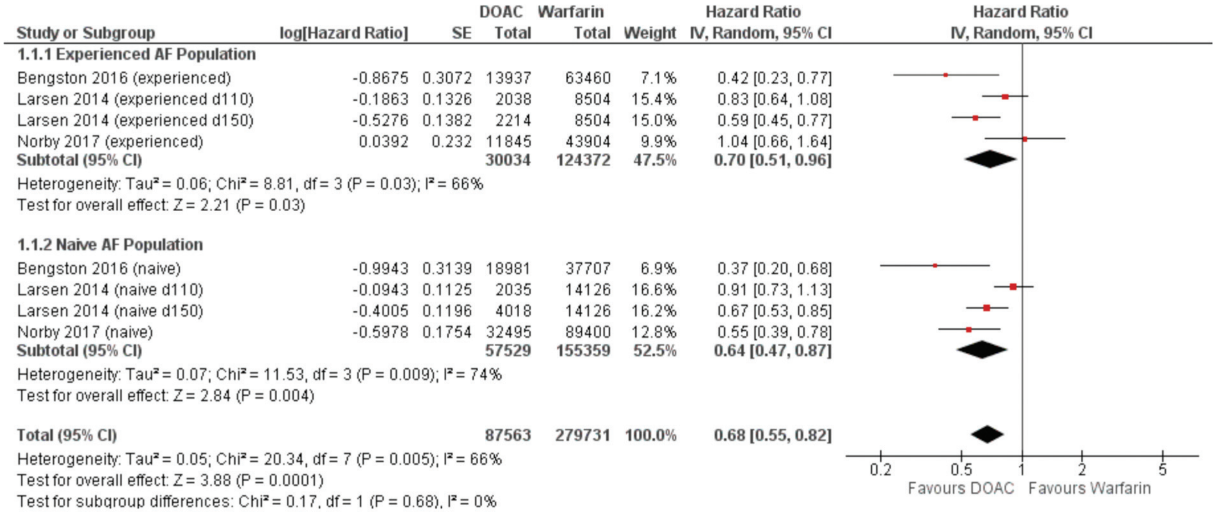

Study or Subgroup	log[Hazard Ratio]	SE	Weight	Hazard Ratio IV, Random, 95% CI
Blin 2018 (dabigatran)	-1.2379	0.3034	12.6%	0.29 [0.16, 0.53]
Blin 2018(dabigatran 110mg)	-0.9416	0.1512	19.4%	0.39 [0.29, 0.52]
Halvorsen 2016 (apixaban)	-0.3011	0.0741	22.4%	0.74 [0.64, 0.86]
Halvorsen 2016 (dabigatran)	-0.3011	0.0584	22.8%	0.74 [0.66, 0.83]
Halvorsen 2016 (rivaroxaban)	0.0953	0.0642	22.7%	1.10 [0.97, 1.25]
**Total (95% CI)**			**100.0%**	**0.64 [0.47, 0.87]**
Heterogeneity: Tau² = 0.11; Chi² = 61.25, df = 4 (P < 0.00001); I² = 93%				
Test for overall effect: Z = 2.87 (P = 0.004)				

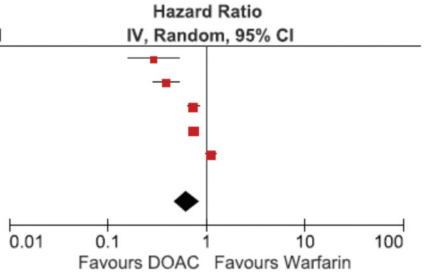

Appendix 5 Forest Plot of Bleeding Risk in AF Patients according to Previous Anticoagulant Exposure

Appendix 6 Forest Plot Comparing Clinically Relevant Nonmajor Bleeding Ratio between DOACs and Warfarin Using Observational Data Only

Study or Subgroup	log[Hazard Ratio]	SE	DOAC Total	Warfarin Total	Weight	Hazard Ratio IV, Random, 95% CI
**1.6.1 Direct Thrombin Inhibitor**						
Blin 2018 (dabigatran)	-1.2379	0.3034	8389	8389	4.8%	0.29 [0.16, 0.53]
Blin 2018(dabigatran 110mg)	-0.9416	0.1512	14440	14440	9.1%	0.39 [0.29, 0.52]
Halvorsen 2016 (dabigatran)	-0.3011	0.0584	7925	11427	12.2%	0.74 [0.66, 0.83]
**Subtotal (95% CI)**			**30754**	**34256**	**26.2%**	**0.46 [0.26, 0.82]**
Heterogeneity: Tau² = 0.22; Chi² = 23.28, df = 2 (P < 0.00001); I² = 91%						
Test for overall effect: Z = 2.65 (P = 0.008)						
**1.6.2 Direct Xa Inhibitor**						
Giugliano 2013 (edoxaban 30mg)	-0.1508	0.0433	7034	7036	12.6%	0.86 [0.79, 0.94]
Giugliano 2013 (edoxaban 60mg)	-0.4155	0.0486	7035	7036	12.5%	0.66 [0.60, 0.73]
Granger 2011 (apixaban)	-0.3857	0.0554	9120	9081	12.3%	0.68 [0.61, 0.76]
Halvorsen 2016 (apixaban)	-0.3011	0.0741	6505	11427	11.8%	0.74 [0.64, 0.86]
Halvorsen 2016 (rivaroxaban)	0.0953	0.0642	6917	11427	12.1%	1.10 [0.97, 1.25]
Patel 2011 (rivaroxaban)	0.0392	0.0408	7131	7133	12.6%	1.04 [0.96, 1.13]
**Subtotal (95% CI)**			**43742**	**53140**	**73.8%**	**0.83 [0.70, 0.99]**
Heterogeneity: Tau² = 0.04; Chi² = 87.37, df = 5 (P < 0.00001); I² = 94%						
Test for overall effect: Z = 2.09 (P = 0.04)						
**Total (95% CI)**			**74496**	**87396**	**100.0%**	**0.73 [0.62, 0.86]**
Heterogeneity: Tau² = 0.05; Chi² = 128.33, df = 8 (P < 0.00001); I² = 94%						
Test for overall effect: Z = 3.82 (P = 0.0001)						
Test for subgroup differences: Chi² = 3.75, df = 1 (P = 0.05), I² = 73.3%						

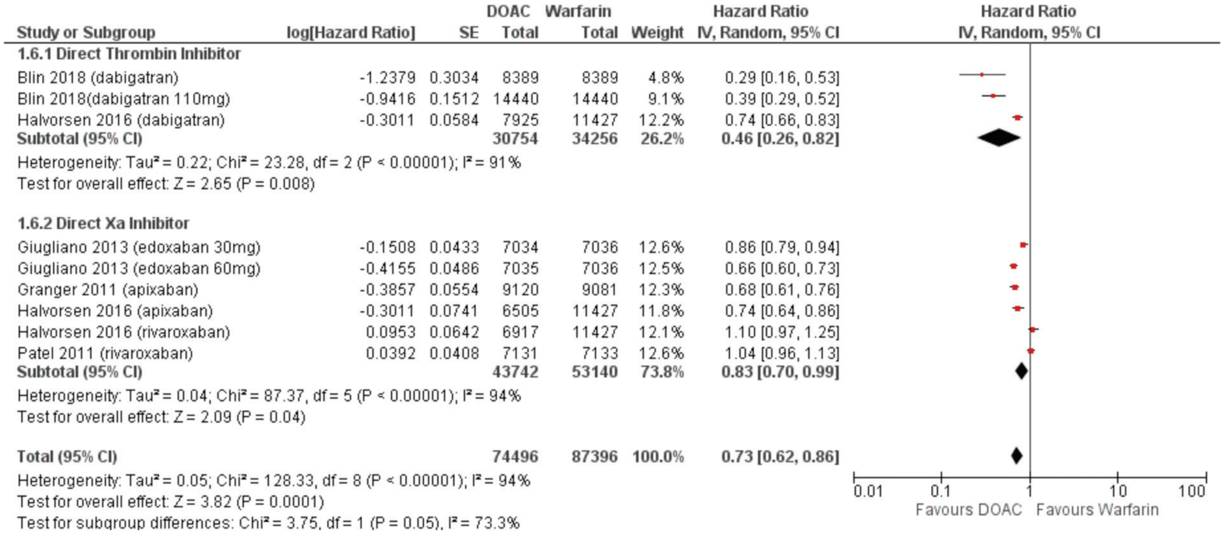

Appendix 7 Forest Plot Comparing Clinically Relevant Nonmajor Bleeding Ratio between DOACs and Warfarin Using Observational and Randomized Trials Data

Study or Subgroup	log[Hazard Ratio]	SE	DOAC Total	Warfarin Total	Weight	Hazard Ratio IV, Random, 95% CI
**1.4.1 Observational Studies**						
Adeboyeje 2017 (apixaban)	-0.6539	0.1213	3669	23431	2.1%	0.52 [0.41, 0.66]
Adeboyeje 2017 (dabigatran)	-0.4005	0.0563	8539	23431	2.4%	0.67 [0.60, 0.75]
Adeboyeje 2017 (rivaroxaban)	0	0.0595	8398	23431	2.4%	1.00 [0.89, 1.12]
Amin 2015 (apixaban)	-0.6931	0.0652	20803	90010	2.4%	0.50 [0.44, 0.57]
Amin 2015 (dabigatran)	-0.2357	0.0691	16731	90010	2.3%	0.79 [0.69, 0.90]
Amin 2015 (rivaroxaban)	0.157	0.0801	52476	90010	2.3%	1.17 [1.00, 1.37]
Blin 2018 (dabigatran)	-1.204	0.2069	8389	8389	1.6%	0.30 [0.20, 0.45]
Blin 2018(dabigatran 110mg)	-0.0408	0.266	14440	14440	1.3%	0.96 [0.57, 1.62]
Bouillon 2015 (dabigatran 110mg)	-0.1625	0.1863	3356	10705	1.7%	0.85 [0.59, 1.22]
Bouillon 2015 (dabigatran)	-0.5276	0.3803	1014	10705	0.9%	0.59 [0.28, 1.24]
Bouillon 2015 (rivaroxaban)	-0.5209	0.3837	731	10705	0.9%	0.59 [0.28, 1.26]
Boullion 2015 (rivaroxaban 10mg)	-0.0726	0.3375	1604	10705	1.0%	0.93 [0.48, 1.80]
Denas 2017	0.0677	0.1358	6740	6740	2.0%	1.07 [0.82, 1.40]
Ellis 2016 (dabigatran 110mg)	0.2927	0.1007	4170	9664	2.2%	1.34 [1.10, 1.63]
Ellis 2016 (dabigatran)	0	0.182	4170	9664	1.8%	1.00 [0.70, 1.43]
Ellis 2016 (rivaroxaban)	-0.3431	0.1489	4170	9664	2.0%	0.71 [0.53, 0.95]
Go 2017 (dabigatran)	-0.1165	0.1082	25289	25289	2.2%	0.89 [0.72, 1.10]
Gorst-Rasmussen 2016 (rivaroxaban 10-15mg)	-0.1054	0.2154	776	11045	1.6%	0.90 [0.59, 1.37]
Gorst-Rasmussen 2016 (rivaroxaban)	0.1655	0.1382	1629	11045	2.0%	1.18 [0.90, 1.55]
Halvorsen 2016 (apixaban)	-0.5798	0.1717	6505	11427	1.8%	0.56 [0.40, 0.78]
Halvorsen 2016 (dabigatran)	-0.4005	0.0825	7925	11427	2.3%	0.67 [0.57, 0.79]
Halvorsen 2016 (rivaroxaban)	-0.1508	0.1198	6917	11427	2.1%	0.86 [0.68, 1.09]
Hernandez 2014 (dabigatran)	0.4574	0.0765	1302	8102	2.3%	1.58 [1.36, 1.84]
Ho 2012 (dabigatran 110mg)	-0.3285	0.6797	122	122	0.4%	0.72 [0.19, 2.73]
Huang 2017 (rivaroxaban)	-0.1865	0.0796	9637	9637	2.3%	0.83 [0.71, 0.97]
Jacobs 2016	-0.3567	0.1048	2627	2627	2.2%	0.70 [0.57, 0.86]
Koretsune 2018 (dabigatran 110mg)	-0.607	0.3046	4606	4606	1.2%	0.54 [0.30, 0.99]
Laliberte 2014 (rivaroxaban)	0.0761	0.2136	3654	14616	1.6%	1.08 [0.71, 1.64]
Larsen 2013 (dabigatran 110mg)	-0.1985	0.168	2739	9289	1.8%	0.82 [0.59, 1.14]
Larsen 2013 (dabigatran)	-0.2614	0.2102	2239	9289	1.6%	0.77 [0.51, 1.16]
Lauffenburger 2015 (dabigatran)	-0.0647	0.0381	21070	43865	2.4%	0.94 [0.87, 1.01]
Lip 2016 (apixaban 5mg)	0.6582	0.278	24102	12713	1.3%	1.93 [1.12, 3.33]
Lip 2016 (apixaban)	-0.642	0.1528	6964	6964	1.9%	0.53 [0.39, 0.71]
Lip 2016 (dabigatran)	-0.367	0.1664	4515	4515	1.9%	0.69 [0.50, 0.96]
Lip 2016 (rivaroxaban)	-0.0202	0.1035	12625	12625	2.2%	0.98 [0.80, 1.20]
Maura 2015 (dabigatran 110mg)	-0.1727	0.1811	5895	11571	1.8%	0.84 [0.59, 1.20]
Maura 2015 (dabigatran)	-0.1626	0.3477	2548	3580	1.0%	0.85 [0.43, 1.68]
Maura 2015 (rivaroxaban)	-0.0171	0.219	2561	5722	1.6%	0.98 [0.64, 1.51]
Russo-Alvarez 2018 (rivaroxaban)	-0.1383	0.5108	472	472	0.6%	0.87 [0.32, 2.37]
Staerk 2016 (apixaban)	-0.6326	0.2277	6899	18094	1.5%	0.53 [0.34, 0.83]
Staerk 2016 (dabigatran)	-0.9943	0.1608	12613	18094	1.9%	0.37 [0.27, 0.51]
Staerk 2016 (rivaroxaban)	-0.4094	0.1986	5693	18094	1.7%	0.66 [0.45, 0.98]
Villines 2015 (dabigatran)	-0.1407	0.0819	12793	12793	2.3%	0.87 [0.74, 1.02]
Vinogradov 2018 (apixaban)	-0.4155	0.1024	10601	70585	2.2%	0.66 [0.54, 0.81]
Vinogradov 2018 (dabigatran)	-0.1446	0.0938	16547	70585	2.2%	0.87 [0.72, 1.04]
Vinogradov 2018 (rivaroxaban)	0.1105	0.0615	5537	70585	2.4%	1.12 [0.99, 1.26]
Yao 2016 (apixaban)	-0.8032	0.1406	7695	7695	2.0%	0.45 [0.34, 0.59]
Yao 2016 (dabigatran)	-0.2312	0.0864	14307	14307	2.3%	0.79 [0.67, 0.94]
Yao 2016 (rivaroxaban)	0.0385	0.0734	16175	16175	2.3%	1.04 [0.90, 1.20]
**Subtotal (95% CI)**			**424979**	**1000686**	**90.3%**	**0.80 [0.73, 0.88]**
Heterogeneity: Tau² = 0.08; Chi² = 387.38, df = 48 (P < 0.00001); I² = 88%						
Test for overall effect: Z = 4.75 (P < 0.00001)						
**1.4.2 Randomized Control Trials**						
Connolly 2012 (betrixaban 80 mg)	-0.4959	0.7322	127	127	0.3%	0.61 [0.15, 2.56]
Giugliano 2013 (edoxaban 30mg)	-0.755	0.0697	7034	7036	2.3%	0.47 [0.41, 0.54]
Giugliano 2013 (edoxaban 60mg)	-0.2231	0.0609	7035	7036	2.4%	0.80 [0.71, 0.90]
Granger 2011 (apixaban)	-0.3711	0.0713	9120	9081	2.3%	0.69 [0.60, 0.79]
Patel 2011 (rivaroxaban)	0.0385	0.0734	7131	7133	2.3%	1.04 [0.90, 1.20]
**Subtotal (95% CI)**			**30447**	**30413**	**9.7%**	**0.72 [0.53, 0.97]**
Heterogeneity: Tau² = 0.10; Chi² = 65.85, df = 4 (P < 0.00001); I² = 94%						
Test for overall effect: Z = 2.14 (P = 0.03)						
**Total (95% CI)**			**455426**	**1031099**	**100.0%**	**0.79 [0.72, 0.86]**
Heterogeneity: Tau² = 0.08; Chi² = 472.97, df = 53 (P < 0.00001); I² = 89%						
Test for overall effect: Z = 5.19 (P < 0.00001)						
Test for subgroup differences: Chi² = 0.47, df = 1 (P = 0.49), I² = 0%						

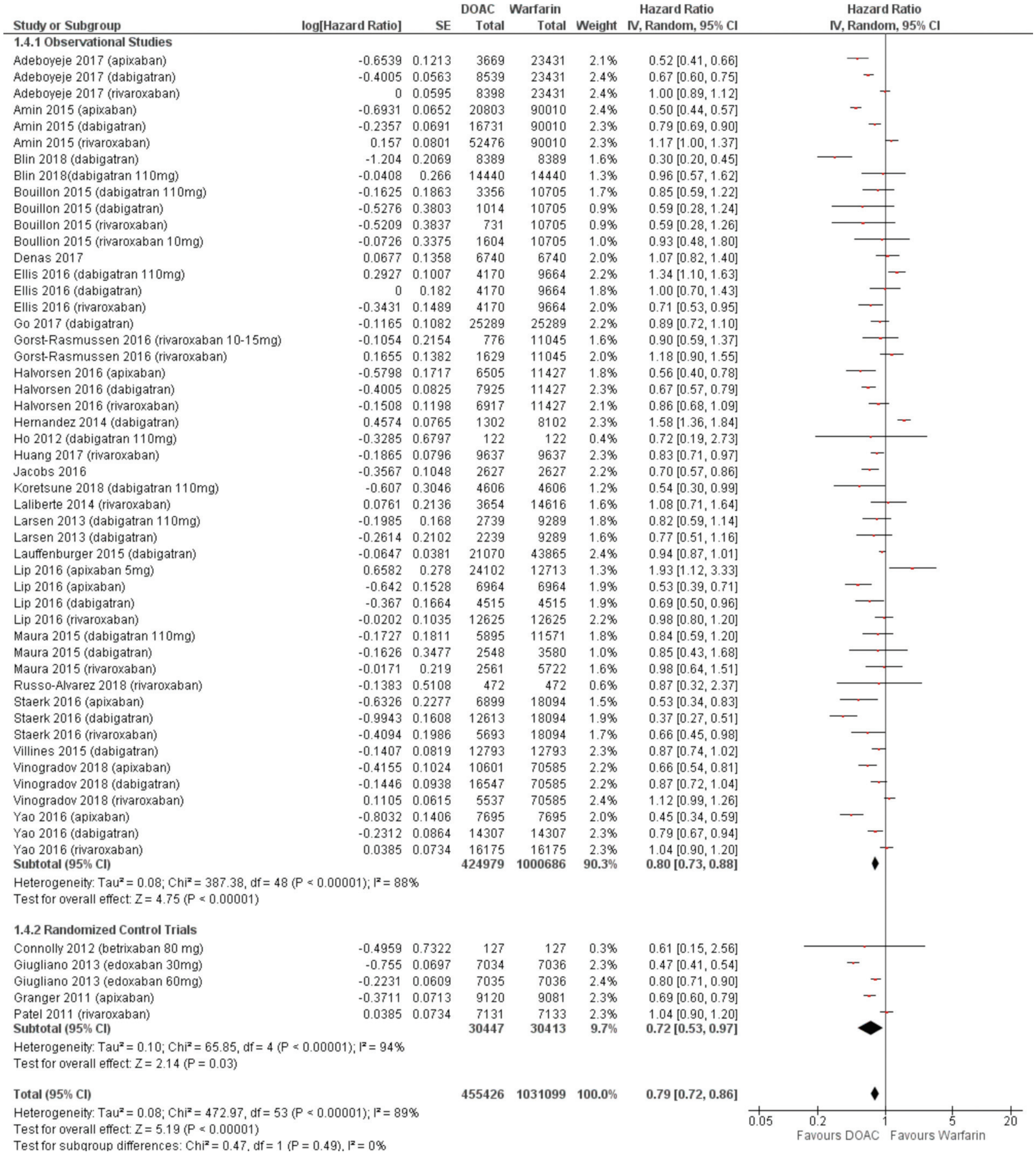

Appendix 8 Forest Plot Comparing Major Bleeding between DOACs and Warfarin Stratified by Study Type

Study or Subgroup	log[Hazard Ratio]	SE	DOAC Total	Warfarin Total	Weight	Hazard Ratio IV, Random, 95% CI
**1.5.1 Pharmaceutical Funding**						
Adeboyeje 2017 (apixaban)	-0.6539	0.1213	3669	23431	2.1%	0.52 [0.41, 0.66]
Adeboyeje 2017 (dabigatran)	-0.4005	0.0563	8539	23431	2.4%	0.67 [0.60, 0.75]
Adeboyeje 2017 (rivaroxaban)	0	0.0595	8398	23431	2.4%	1.00 [0.89, 1.12]
Amin 2015 (apixaban)	-0.6931	0.0652	20803	90010	2.4%	0.50 [0.44, 0.57]
Amin 2015 (dabigatran)	-0.2357	0.0691	16731	90010	2.3%	0.79 [0.69, 0.90]
Amin 2015 (rivaroxaban)	0.157	0.0801	52476	90010	2.3%	1.17 [1.00, 1.37]
Blin 2018 (dabigatran)	-1.204	0.2069	8389	8389	1.6%	0.30 [0.20, 0.45]
Blin 2018(dabigatran 110mg)	-0.0408	0.266	14440	14440	1.3%	0.96 [0.57, 1.62]
Connolly 2012 (betrixaban 80 mg)	-0.4959	0.7322	127	127	0.3%	0.61 [0.15, 2.56]
Ellis 2016 (dabigatran 110mg)	0.2927	0.1007	4170	9664	2.2%	1.34 [1.10, 1.63]
Ellis 2016 (dabigatran)	0	0.182	4170	9664	1.8%	1.00 [0.70, 1.43]
Ellis 2016 (rivaroxaban)	-0.3431	0.1489	4170	9664	2.0%	0.71 [0.53, 0.95]
Giugliano 2013 (edoxaban 30mg)	-0.755	0.0697	7034	7036	2.3%	0.47 [0.41, 0.54]
Giugliano 2013 (edoxaban 60mg)	-0.2231	0.0609	7035	7036	2.4%	0.80 [0.71, 0.90]
Granger 2011 (apixaban)	-0.3711	0.0713	9120	9081	2.3%	0.69 [0.60, 0.79]
Halvorsen 2016 (apixaban)	-0.5798	0.1717	6505	11427	1.8%	0.56 [0.40, 0.78]
Halvorsen 2016 (dabigatran)	-0.4005	0.0825	7925	11427	2.3%	0.67 [0.57, 0.79]
Halvorsen 2016 (rivaroxaban)	-0.1508	0.1198	6917	11427	2.1%	0.86 [0.68, 1.09]
Koretsune 2018 (dabigatran 110mg)	-0.607	0.3046	4606	4606	1.2%	0.54 [0.30, 0.99]
Laliberte 2014 (rivaroxaban)	0.0761	0.2136	3654	14616	1.6%	1.08 [0.71, 1.64]
Lip 2016 (apixaban 5mg)	0.6582	0.278	24102	12713	1.3%	1.93 [1.12, 3.33]
Patel 2011 (rivaroxaban)	0.0385	0.0734	7131	7133	2.3%	1.04 [0.90, 1.20]
Villines 2015 (dabigatran)	-0.1407	0.0819	12793	12793	2.3%	0.87 [0.74, 1.02]
**Subtotal (95% CI)**			**242904**	**501566**	**45.1%**	**0.77 [0.67, 0.88]**
Heterogeneity: $Tau^2 = 0.09$; $Chi^2 = 254.52$, df = 22 (P < 0.00001); $I^2 = 91\%$						
Test for overall effect: Z = 3.74 (P = 0.0002)						
**1.5.2 Research Grants and Non-For-Profit**						
Bouillon 2015 (dabigatran 110mg)	-0.1625	0.1863	3356	10705	1.7%	0.85 [0.59, 1.22]
Bouillon 2015 (dabigatran)	-0.5276	0.3803	1014	10705	0.9%	0.59 [0.28, 1.24]
Bouillon 2015 (rivaroxaban)	-0.5209	0.3837	731	10705	0.9%	0.59 [0.28, 1.26]
Boullion 2015 (rivaroxaban 10mg)	-0.0726	0.3375	1604	10705	1.0%	0.93 [0.48, 1.80]
Denas 2017	0.0677	0.1358	6740	6740	2.0%	1.07 [0.82, 1.40]
Go 2017 (dabigatran)	-0.1165	0.1082	25289	25289	2.2%	0.89 [0.72, 1.10]
Gorst-Rasmussen 2016 (rivaroxaban 10-15mg)	-0.1054	0.2154	776	11045	1.6%	0.90 [0.59, 1.37]
Gorst-Rasmussen 2016 (rivaroxaban)	0.1655	0.1382	1629	11045	2.0%	1.18 [0.90, 1.55]
Hernandez 2014 (dabigatran)	0.4574	0.0765	1302	8102	2.3%	1.58 [1.36, 1.84]
Ho 2012 (dabigatran 110mg)	-0.3285	0.6797	122	122	0.4%	0.72 [0.19, 2.73]
Huang 2017 (rivaroxaban)	-0.1865	0.0796	9637	9637	2.3%	0.83 [0.71, 0.97]
Jacobs 2016	-0.3567	0.1048	2627	2627	2.2%	0.70 [0.57, 0.86]
Larsen 2013 (dabigatran 110mg)	-0.1985	0.168	2739	9289	1.8%	0.82 [0.59, 1.14]
Larsen 2013 (dabigatran)	-0.2614	0.2102	2239	9289	1.6%	0.77 [0.51, 1.16]
Lauffenburger 2015 (dabigatran)	-0.0647	0.0381	21070	43865	2.4%	0.94 [0.87, 1.01]
Lip 2016 (apixaban)	-0.642	0.1528	6964	6964	1.9%	0.53 [0.39, 0.71]
Lip 2016 (dabigatran)	-0.367	0.1664	4515	4515	1.9%	0.69 [0.50, 0.96]
Lip 2016 (rivaroxaban)	-0.0202	0.1035	12625	12625	2.2%	0.98 [0.80, 1.20]
Maura 2015 (dabigatran 110mg)	-0.1727	0.1811	5895	11571	1.8%	0.84 [0.59, 1.20]
Maura 2015 (dabigatran)	-0.1626	0.3477	2548	3580	1.0%	0.85 [0.43, 1.68]
Maura 2015 (rivaroxaban)	-0.0171	0.219	2561	5722	1.6%	0.98 [0.64, 1.51]
Russo-Alvarez 2018 (rivaroxaban)	-0.1383	0.5108	472	472	0.6%	0.87 [0.32, 2.37]
Staerk 2016 (apixaban)	-0.6326	0.2277	6899	18094	1.5%	0.53 [0.34, 0.83]
Staerk 2016 (dabigatran)	-0.9943	0.1608	12613	18094	1.9%	0.37 [0.27, 0.51]
Staerk 2016 (rivaroxaban)	-0.4094	0.1986	5693	18094	1.7%	0.66 [0.45, 0.98]
Vinogradov 2018 (apixaban)	-0.4155	0.1024	10601	70585	2.2%	0.66 [0.54, 0.81]
Vinogradov 2018 (dabigatran)	-0.1446	0.0938	16547	70585	2.2%	0.87 [0.72, 1.04]
Vinogradov 2018 (rivaroxaban)	0.1105	0.0615	5537	70585	2.4%	1.12 [0.99, 1.26]
Yao 2016 (apixaban)	-0.8032	0.1406	7695	7695	2.0%	0.45 [0.34, 0.59]
Yao 2016 (dabigatran)	-0.2312	0.0864	14307	14307	2.3%	0.79 [0.67, 0.94]
Yao 2016 (rivaroxaban)	0.0385	0.0734	16175	16175	2.3%	1.04 [0.90, 1.20]
**Subtotal (95% CI)**			**212522**	**529533**	**54.9%**	**0.81 [0.73, 0.91]**
Heterogeneity: $Tau^2 = 0.06$; $Chi^2 = 177.24$, df = 30 (P < 0.00001); $I^2 = 83\%$						
Test for overall effect: Z = 3.74 (P = 0.0002)						
**Total (95% CI)**			**455426**	**1031099**	**100.0%**	**0.79 [0.72, 0.86]**
Heterogeneity: $Tau^2 = 0.08$; $Chi^2 = 472.97$, df = 53 (P < 0.00001); $I^2 = 89\%$						
Test for overall effect: Z = 5.19 (P < 0.00001)						
Test for subgroup differences: $Chi^2 = 0.39$, df = 1 (P = 0.53), $I^2 = 0\%$						

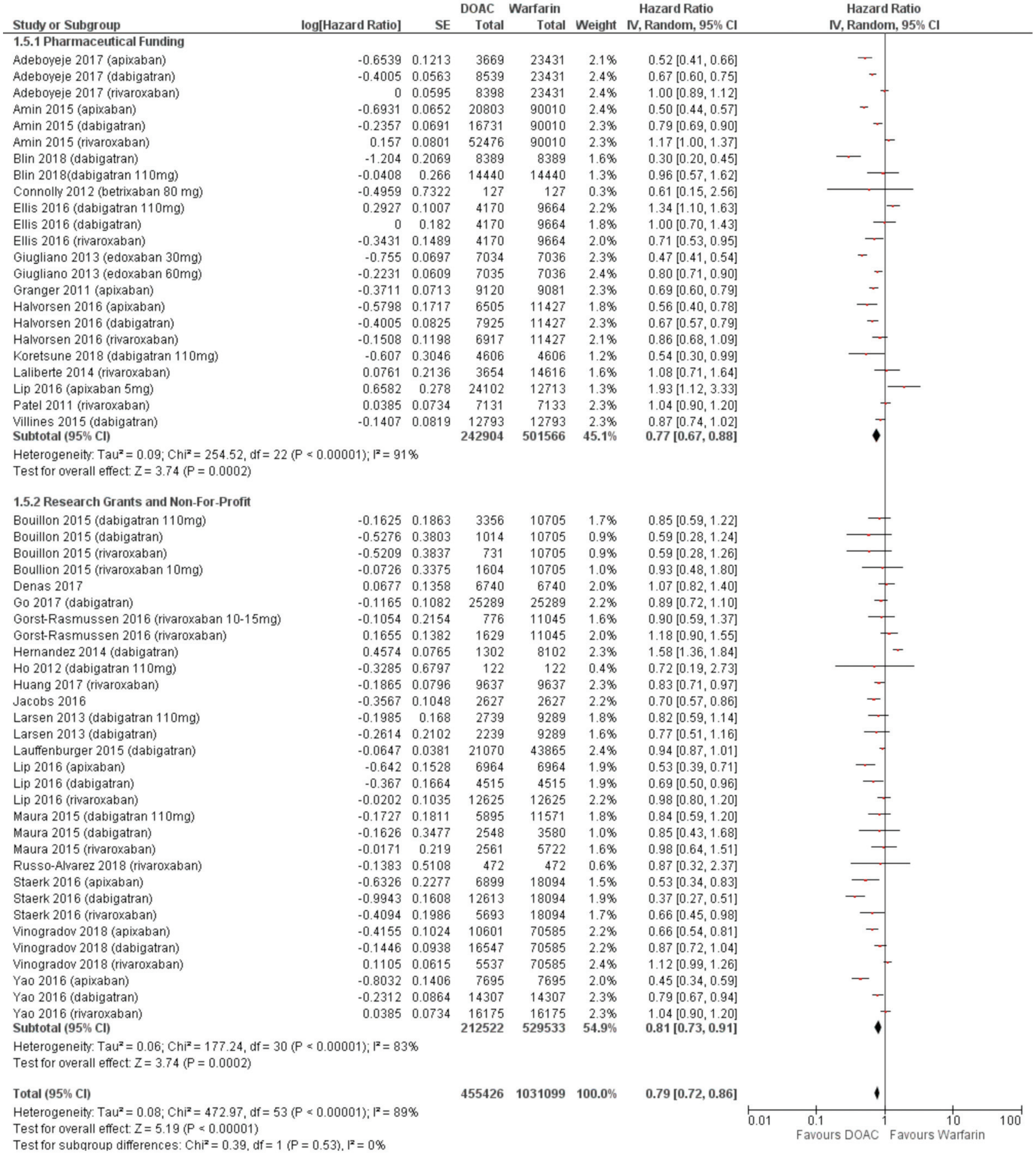

Appendix 9 Forest Plot Comparing Major Bleeding between DOACs and Warfarin Stratified by Funding Type
